# Fine mapping of the GLC1K juvenile primary open-angle glaucoma locus and exclusion of candidate genes

**Published:** 2008-07-21

**Authors:** A. Sud, E.A. Del Bono, J.L. Haines, J.L. Wiggs

**Affiliations:** 1 Department of Ophthalmology, Harvard Medical School, Massachusetts Eye and Ear Infirmary, Boston, MA; 2 Center for Human Genetics Research, Vanderbilt School of Medicine, Nashville, TN

## Abstract

**Purpose:**

Primary open-angle glaucoma is a leading cause of blindness worldwide. We previously identified a region on chromosome 20p12 associated with juvenile-onset primary open-angle glaucoma (JOAG) that was designated GLC1K. The aim of this study is to refine the boundaries of the GLC1K region and to screen selected candidate genes located within the refined region for biologically significant mutations.

**Methods:**

Four JOAG families (44 individuals) with linkage to GLC1K were used for this study. Informative single nucleotide polymorphism (SNP) markers located throughout the previously defined region were used for haplotype analysis. Four candidate genes within the refined region were screened for biologically significant mutations using direct genomic sequencing: bone morphogenetic protein 2 (*BMP2*); phospholipase C beta 1 (*PLCB1*); phospholipase C beta 4 (*PLCB4*); and BTB POZ domain containing 3 (*BTBD3*).

**Results:**

Haplotype analysis identified a new critical interval of 12.7 Mb using a combination of SNPs and microsatellite markers. This analysis extended the region of GLC1K from D20S846 to rs6081603 in affected individuals, and the region was further reduced to 9 Mb if unaffected recombinant individuals were included in the analysis. Biologically significant DNA sequence variants were not identified in the *BMP2*, *PLCB1*, *PLCB4*, or *BTBD3* genes in these families.

**Conclusions:**

Using recombinant breakpoint mapping and haplotypes based on a combination of SNP and microsatellite markers, the GLC1K region has been reduced to a maximum of 12.7 Mb and a minimum of 9 Mb. Four genes that are located within the refined region with attractive ocular expression and function have been excluded as causative genes for JOAG.

## Introduction

Glaucoma is the principal cause of optic nerve degeneration and the second leading cause of blindness worldwide. The disease is predicted to affect more than 50 million people including more than 3 million people in the United States by the year 2020 [1,2]. All forms of glaucoma are characterized by a loss of retinal ganglion cells, leading to optic nerve degeneration and corresponding visual field defects [3]. Unless the disease is identified and therapeutic intervention is begun at an early stage, individuals with glaucoma develop irreversible blindness. Primary open-angle glaucoma (POAG; OMIM 137760) is the most common form of glaucoma and is associated with an open chamber angle and normal-appearing trabecular meshwork. The prevalence of POAG is low in persons under the age of 50 but increases significantly after the age of 70 [4-6]. Juvenile open-angle glaucoma (JOAG) is a rarer subset of POAG that develops before the age of 40 [7].

A family history of glaucoma is widely recognized as a major risk factor for the disease, suggesting that specific gene defects contribute to the pathogenesis [8]. Indeed, both Mendelian and non-Mendelian forms of inheritance have been described; the more common adult-onset form of POAG has heritability consistent with that of a complex trait while JOAG exhibits autosomal dominant inheritance [9,10].

Linkage approaches using large pedigrees affected by POAG have lead to the identification of 14 major genetic loci for adult-onset POAG (GLC1A-GLC1N) [11-14] and three genes that contribute to POAG have been identified. Myocilin (*MYOC*, GLC1A, OMIM 601652) is responsible for 20% of patients with JOAG [15] and 3%–5% of POAG, optineurin (*OPTN*, GLC1E, OMIM 602432) [16] is primarily associated with low tension glaucoma, and WD repeat domain 36 (*WDR36*, GLC1G, OMIM 609669) [17] may be a modifying factor that can influence the severity of disease [18]. None of the disease-associated DNA sequence variants in any of these genes are responsible for a significant fraction of the disease in the POAG population [16-25]. These results highlight the multifactorial inheritance of POAG and point to the existence of additional loci and genes that can contribute to this complex disease [10,11].

Six of the open-angle glaucoma loci are primarily associated with early onset open-angle glaucoma or JOAG: GLC1A, GLC1J, GLC1K, GLC1M, GLC1N, and a novel region on 2p15–16 that partially overlaps with GLC1H [12,13,15,26-28]. We previously performed a genome-wide scan using 25 JOAG families that identified two novel JOAG loci on 9q22 (GLC1J) and 20p12 (GLC1K) [26]. In our previous study, haplotype analysis of 15 families identified seven families with consistent linkage to the GLC1J region, five families with consistent linkage to the GLC1K region, and three families with consistent linkage to both regions. Recombination events identified a 9 cM region on chromosome 9 between markers D9S1841 and D9S271 (GLC1J) and a 46 cM region on chromosome 20 between markers D20S846 and D20S891 (GLC1K). The purpose of the current study is to saturate the GLC1K region with single nucleotide polymorphism (SNP) markers making further definition of the boundaries of the critical recombinant region possible and to screen candidate genes located in the refined region for biologically significant mutations.

## Methods

### Families

This study adhered to the tenets of the Declaration of Helsinki and has been reviewed and approved by the Institutional Review Board of the Massachusetts Eye and Ear Infirmary. Four JOAG families (44 individuals) with previously demonstrated linkage to GLC1K were used for this study. All of these families demonstrated inheritance patterns consistent with autosomal dominant inheritance and had sufficient size and structure that haplotype analysis could be performed using all four of the original parental chromosomes. All of the sampled family members (affected and unaffected) who entered into this study underwent a complete ocular examination including gonioscopy, tonometry, and fundoscopy. All affected individuals also had visual field testing using the automated Humphrey perimeter. Affected individuals met the following three criteria: 1) intraocular pressure measured by applanation tonometry in both eyes was greater than 22 mmHg or greater than 19 mmHg on two glaucoma medications, 2) there was glaucomatous optic neuropathy in both eyes, and 3) visual field loss was consistent with optic nerve damage in at least one eye. Juvenile open-angle glaucoma (JOAG) is defined as patients who meet these criteria and have an age of onset before the age of 40 while adult onset primary open-angle glaucoma patients meet the above criteria and have an age of onset after the age of 40 [7]. Glaucomatous optic neuropathy was defined as a cup to disk ratio higher than 0.7 or focal loss of the nerve fiber layer associated with a visual field defect. The affected family members had normal appearing angle structures on gonioscopy, and none of the affected family members had any evidence of secondary glaucomas including pigment dispersion, anterior segment dysgenesis, and corneal abnormalities.

### Genotyping

Genomic DNA was prepared from blood samples from family members using previously described techniques [18]. SNP genotyping was performed using a quantitative polymerase chain reaction (PCR) approach (TaqMan Assay; Applied Biosystems, Foster City, CA) according to the manufacturer’s instructions. The reverse transcription polymerase chain reaction (RT–PCR) amplification of genomic DNA was performed in a 96 well plate with a sequence detection system (ABI Prism©7000 Sequence Detection System; Applied Biosystems Inc., Foster City, CA). The thermal cycler (model 2720, Applied Biosystems Inc., Foster City, CA) was set at the following parameters: 50 °C for 2 min, 95 °C for 10 min, 92 °C for 15 s, and 58 °C for 1 min for a total of 60 cycles. Microsatellite genotypes had been previously obtained [26].

### Haplotype analysis

Forty SNPs with minor allele frequency greater than 40% were chosen at approximately 100,000 base pair intervals throughout the previously defined GLC1K region [29]. Alleles from informative SNP markers were added to previously established microsatellite-based haplotypes [26]. Haplotypes were deduced initially using the Simwalk 2 program [30]. These haplotypes were confirmed by visual inspection, and ambiguities in transmission were resolved where possible.

### DNA sequencing

All exons and 100 base pairs of the flanking intron sequence were sequenced for bone morphogenetic protein 2 (*BMP2*); phospholipase C beta 1 (*PLCB1*); phospholipase C beta 4 (*PLCB4*); and BTB POZ domain containing 3 (*BTBD3*) using nested PCR strategies for amplification. Oligonucleotides for amplification and sequencing were selected using Primer3 software (provided by Massachusetts Institute of Technology, Cambridge, MA) and were located at least 40 bp from each exon’s splice site. Primer sequences are presented in Table 1. PCR was performed in a thermal cycler in a total volume of 25 μl containing 50 ng genomic DNA; 1.5 mM MgCl_2_; 200 μM each of dATP, dCTP, dGTP, and dTTP; 100 ng forward PCR primer, 100 ng reverse PCR primer; 20 mM Tris-HCl (pH 8.4); 50 mM KCl; and 0.5 U Taq DNA polymerase (Platinum Taq; Invitrogen-Life Technologies, Rockville, MD). Cycling conditions were as follows: an initial denaturing step of 5 min at 94 °C; 35 cycles of denaturation (94 °C for 45 s), annealing (primer-specific annealing temperature for 60 s), elongation (72 °C for 45 s), and a final elongation step of 5 min at 72 °C. Amplified genomic DNA was directly sequenced using sequencing chemistries (BIGDYE version 3.1; Applied Biosystems Inc.) and an automated sequencer (model 3100; Applied Biosystems Inc.). Sequences were analyzed using sequencer software (Gene Codes Corp., Ann Arbor, MI) and compared to the gene sequence in the public database [31,32].

**Table 1:** Oligonucleotide primers.

**Gene**	**Exon**	**Forward primer**	**Reverse primer**	**Sequence primer**
*BMP2*	1A	ggaacttgggacccttcatt	agtgcctgcgatacaggtct	ttgagcttcggtcggtctta
	1B	cttctagcgttgctgcttcc	ccaccacacaagcagtgagt	tcctgagcgagttcgagttg
	2A	atcaaatcccacgatgaggt	cacttccaccacgaatccat	tcaaacgtcattacttggct
	2B	aaacctgcaacagccaactc	ctcgtcaaggtacagcatcg	ggtgaatcagaatgcaagca
	2C	gagacaccctttgtacgtgga	tacttcatgtgctggggttg	atcatctgaactccactaat
*PLCB1*	1	ggcttctcttcgccttcc	agtgcgcccattgcataac	tccggagcagagaaaggagc
	2	ggaaagaacctcagttacataatgg	gactttgaaacgtcctaagtcct	tgactactttggagaatctg
	3	tgatttaggaatggcttgtagga	gggcatactggctattgtgtt	cttcagtcaagtctcaagat
	4	tacctctgggcaagttgctt	gcacttatacttggcagctgaa	cattctaactcaacagaagt
	5	aaagaacatctcctttggaaaa	gtgcagcttggaggaaagag	gaactaatgtgtcttcctgg
	6	ggcttctggaatgctgctaa	gcatttccacactcccactt	ttaatctgggtgccaactgt
	7	atgcaaatgcatggaggaa	ttcctctccgatgataatcca	tgagttggcatcatctgtta
	8	ccctaaagccagtgtccaaa	gggcaagattgtgtgatcct	acgctacggttgacctcata
	9	cggtagcaattgcacaagat	ccgaaaggaatgccttgtaa	aggtatagaaattatggtgg
	10	atttggtggggctcatgtaa	agagttctcagaggcacttcg	taccttggacactacaagat
	11	aaattgagccattaccttctgg	gcctctgaaattgcaaagatg	taactgctgcagctcttaga
	12	tccagaatcaagagtcagaattagc	gatcctacaagtccaacggataa	gatacaattctctgaatgga
	13	ttcgctgtccatctgtgtatg	caccaactgcattaatcttacca	agttaagataaagatccagg
	14A	ttgggtttctatgttccattg	ttccttctgatgacttgtgtga	tatggtcttcttcatgtaga
	14B	tgagcagtgttgaaggtaggg	ggatggtctcgaactcctga	tagctggaatctggagttcc
	15	tgttttcaatgccacgtgtt	tcttctccggattgtggaac	cttccaaggaaatctgtaat
	16	aggtgttgctgttgcctttc	tgtgtaaagtaacaaggcagtcaa	tctgcatgcttgaatcatgt
	17	tctgacccaagtagcaaacg	acacagagcttggtggtgtg	cctcaacttcactgaatatt
	18	tatcccaaagtagcccaacg	ttcccttcattgaaatttgctt	ggtagcttcttagttactat
	19	tctgtgtttcctgcatttctaca	gagaacacacggacccctta	aagtggactgggatcaactt
	20	tgggtgaggacatagaacca	ccgtccatttgaggcatact	ttgctgaagagcatttgtgg
	21	caagatcacgccactgcac	ctaaatctcatcgcccttgtt	gtcctgattgaataatacct
	22	tgacatgtgatttggaattatcttt	cgaaagtaggggtgattctga	atcacatagatgcgcataat
	23	aatgactgggtggatggatg	tgctattccttctcccaagg	atggtcagatagatgagtaa
	24	cgggcatagacataggttgg	ggagctcctcctaagtccaca	tggctcaacactatgctgtt
	25	actgcacctggcctaatgtt	ttttatgccagtagggttctagc	agagatttagattcaaggcc
	26	cccaggtagagctgacatcaa	aaagctatggagaacggaggt	ttgctcttaaactctagctg
	27	ttcagtctctgggattggaga	tctcaagttggtgccagtca	aggaattctggtgaataacc
	28/29	tggttctaaattgtcctcattgc	cacgaaggagtctttgactgg	catccatcacagatattcta
	30	gcaacagagcgaaaccttgt	gcaattcaacttgcagaaatgt	aatgttgagagttctgtaac
	31	agtgcagtccattgatcacc	caccaggtgatgataaatgctta	atgtaagccatagatctgaa
	32	cctctataggtgaccagtgaatga	cttctcgggaagatggtcct	gtatatctctttcagagagt
*PLCB4*	1	cccaaatgatcacctctggt	gcctcttttcaacaactccaa	gcgaagtgattaaccatcct
	2	aacctatcctagagcaagatgtttt	gaggataatgtccttaatcatcactg	gtttagaagctcagcaatat
	3	gcactgccttcatcatcaga	gccttttattagggtctgcttg	agcagtttgcctgtatcata
	4	gaatgcaccactgttcacca	cccacaggagtgacatgcta	gaataatgctaggtgtctgg
	5	atggcacaatagatggcaca	gaggaaggaaccacctagcc	agatggctgtgtgacaggaa
	6	caaccctaaattcatcttatagcaca	tttgccacctattttgacca	taacagaattgaagctgctc
	7	ctaaccagctctgggaacca	agctggccttcatttccttt	tagtctcactctccatagta
	8	tagtgagatggcctggcagt	aaagttccaggtgctgcaat	gcaggaatcagagatgaaga
	9	gggagtgttttccttctgttg	gtgcaaagccaatgtgaaga	agaattcaccactacttcag
	10	ttaaggagaaaggcaggaagg	gtcatcaatccatggctcag	cagtgaggtcctatctttgt
	11	gacagatgaaagttcttgggaaa	caacgttaaatgaccatgataatcc	gctgcctcagcttccatgaa
	12	tgataataccaaccagatgaggaa	tgaaggcataatgcttgagaaa	ttaacctgtatatgccatga
	13	ccacagggaactcaaccttt	gtgcccttgctggagaatta	ccaggtctgaattgctggaa
	14	tctcacattagcattaagaggacaa	tgatgtaagctttccgtaccaa	gtgagaatctttaattgcct
	15	gctctgcacacctctggaa	tgtggcaatcttgctctgaa	gtttgcagcactagtctaag
	16	tcacgaggactgaacctcct	attatgggcttgagccactg	tgtcgatctcaaggcctagg
	17	ccttgacgttgaataggtaagagtt	tggccctgactaatgagtaattt	tactgcatatatcaatgtcc
	18	ctccttttgcattattcagagc	ccacataccaaggtggcttc	caacagacactaacttctta
	19	tctccatccacaggatgtca	gccatggtctttcccacata	gctactgacatgataatgta
	20	catggcagctgtgacaatct	ttgctttgccatatgtctgc	gaactgtgatcttaagcatc
	21	tgccttaaatagaccaagcaga	tcccagacataaaatcctcca	gcctaggagcaatctgatac
	22	ggcacattctcttccatcct	aagttgtgagcagcacatgg	agctcctgtgttacagatgg
	23	gaaggtagaacatctgcttgaaa	tgttgtcggttgaataagtgc	tgcctatagtagttgttcga
	24	tcttgggcactgtagctcat	ggcagcattagcaggaataga	ggaatgcagatggatcatgg
	25	agctgtgccttcctcagtgt	gccactgatgatgaagtggat	caggtatccaggattgtagt
	26	ctttccttggctggatcctt	acggcctgtccaggtaacta	gtatggtagaagtttactgg
	27	tgccctcaatcaatcacttg	tgcttcaagtaagcctttgg	tcgatgagatttcgatatgt
	28	aaggatagaagacttaaagcagatga	gcactcccaaaccgattcta	ctcaccaagtgtaatcattg
	29	tgttgaatgggagatctgga	ccttgacctaaacagcagca	gtctgtactgttctcagatt
	30	aaagaccagtgttgcccaat	ccagatcagctgcaggctac	gagccatgacatctcatatt
	31	aacttcctgcttcccattga	tcagccatatgatgagatacacaa	tgattcaccaattaagttgg
	32	tgagaggagtgagcctagtca	gccacaagtgttttgctgtc	agtgcatgacattaagtaca
	33	tgaatcaggaagaatacacatgg	ggagcagagaagctggaatg	cttctctggacattatatgt
	34	ggtttagtcatgcgtaagtcctc	atatgcaagccactggtca	tagcaggaaagatcttccca
	35	taagaaggtcccaggcaatg	cagacattccaagtctctctgg	tagcaaggatgtggtgacag
*BTBD3*	1A	caggaatcatgatggggata	gcaacttgctgctgttgcta	tcagttaacctcttagcccg
	1B	tgatgcttccagagacggta	gcttctgggaaaagaggaaa	gcagcaagttgccaccagtt
	2	cattcaagtgacagggttgtct	aaaattaagcgtggccaaag	tgccttgaagttactggact
	3	catggcaagctaccctgtgt	cccaaattctacgtcaaggtg	gcagtagattttaggtacgc
	4A	gaggctagaatggtagcacaaag	acttcagcccagttgagagc	gaatgttcctttactctgca
	4B	ctgttcgaggagccagacct	cctgccgcttaagttcaatc	tgattgatgcccaggctgag
	4C	gtcaccgtttccagtcgtgt	aattaccggcactgatcaca	agcatccagtttgcagttga

Oligonucleotide primers used to PCR amplify each of the indicated exons of *BMP2*, *PLCB1*, *PLCB4*, and *BTBD3*, along with the primer sequence used to sequence each PCR product.

## Results

### Reduction of the GLC1K critical interval

**Figure 1:**
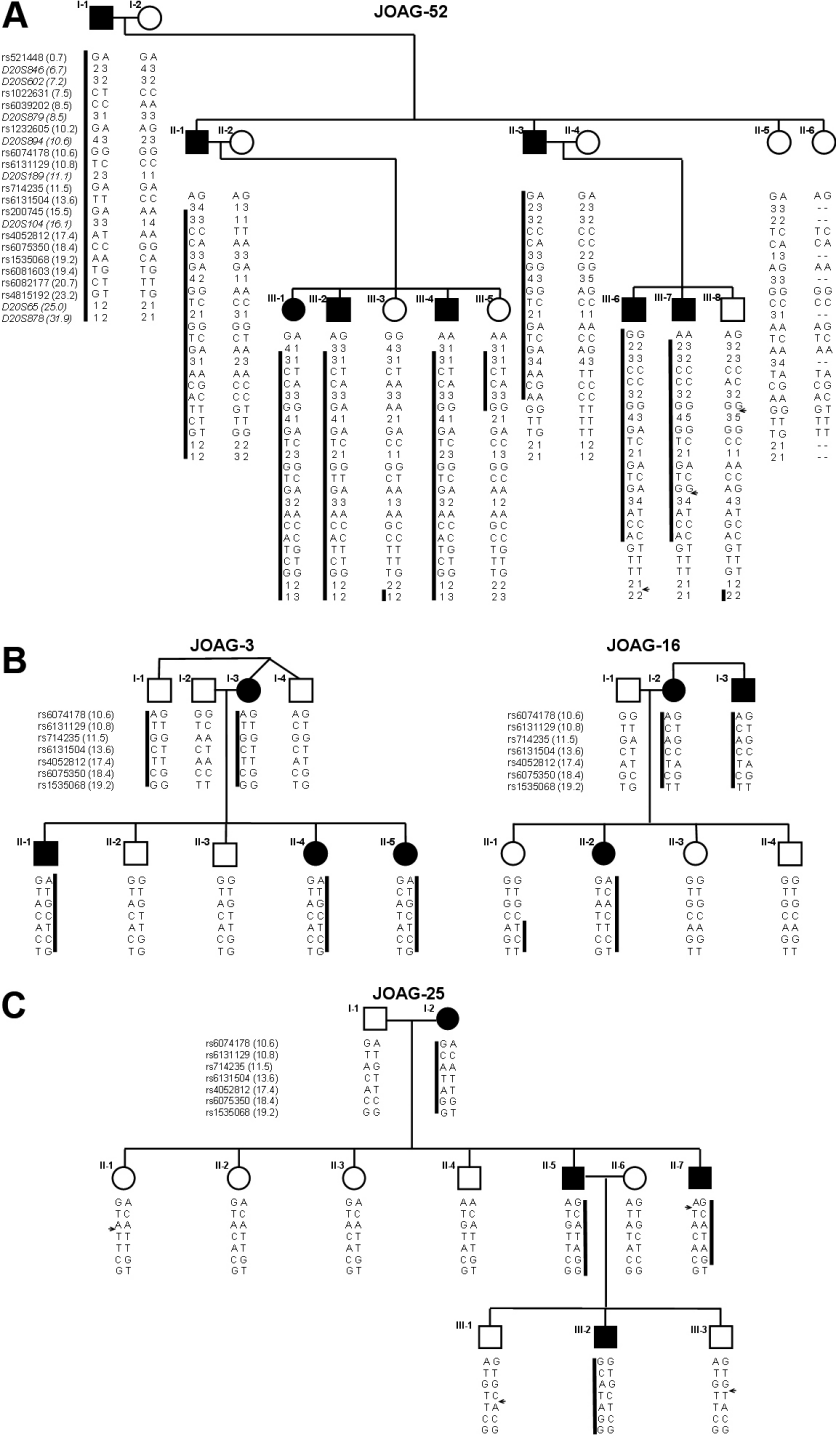
*GLC1K* haplotype analysis in JOAG families. Haplotypes consisting of alleles for microsatellite repeat markers and SNPs are shown under each individual in the pedigrees. The boxed regions indicate the haplotype that segregates with the affected status. The small arrow identifies the location of a recombination event on the non-disease chromosome. The UCSC genome browser [27] was used to determine the location of the markers.

**Table 2:** DNA sequence variants in candidate genes located within the GLC1K refined region.

**Gene (Mb)**	**Ocular expression**	**Function**	**Sequence variant**	**Family**	**Individual**
*BMP2* (6.7)	Lens, fetal eye	Member of TGFB superfamily	c.1946 C>T D387D rs13037675	JOAG-25	III-2
			c.2220 A>C 3′ UTR	JOAG-52	II-1
*PLCB1* (8.6)	Retina, fetal eye	Intracellular transduction of extracellular signals	c.105 C>T D34D rs16994453	JOAG-25	III-2
			c.2085 C>T G694G rs3761170	JOAG-25	III-2
			c.2202 A>G V733V rs8118206	JOAG-52	II-1
			c.2568 C>T A855A rs2076413	JOAG-25	III-2
			c.2991 A>G A996A rs2235613	JOAG-52	II-1
*PLCB4* (9.4)	Ciliary body, trabecular meshwork, retina, retinal pigment epithelium	Intracellular transduction of extracellular signals in the retina	c.197 A>GA21T rs6077510	JOAG-52 JOAG-25	II-1, III-2
*BTBD3* (11.6)	Retina, iris, fetal eye	Protein–protein interaction; DNA binding protein	c.909 G>A A303A rs35364034	JOAG-52	II-1
			IVS2+12G>A	JOAG-52	II-1

The DNA sequence variants identified by genomic sequencing of all exons and flanking intron sequences are shown for each candidate gene. Expression data are from the Stanford SOURCE database.

The GLC1K region was previously identified as a glaucoma gene locus by a microsatellite-based genome scan using a population of 25 multi-generational JOAG pedigrees (overall multipoint LOD score of 4.0) [26]. Haplotypes that were based on the microsatellite repeat markers used in the scan identified key recombination events in affected members in pedigree JOAG-52, a three-generation family with sufficient size and structure to establish independent linkage to the GLC1K region (maximum LOD score=3.2). These recombination breakpoints defined a 46 cM region extending from marker D20S846 to marker D20S891 in affected individuals and a 23 cM region extending from marker D20894 to marker D20878 if unaffected individuals were included in the analysis. To reduce the size of the critical region, 40 SNPs (single nucleotide polymorphisms) with minor allele frequencies of at least 40% were selected at approximately 100 Kb intervals throughout the previously defined GLC1K region [29]. Fifteen SNPs were informative for haplotype analysis, and alleles from these SNPs were evaluated for segregation in all members of pedigree JOAG-52 (Figure 1A). Haplotype analysis using the previous microsatellite alleles as well as the added SNP alleles identified recombination breakpoints that defined a new critical interval of 12.7 Mb, extending from marker D20S846 to marker rs6081603, a reduction of approximately 26 Mb. The critical recombination events defining the 12.7 Mb region occurred in individuals II-1 and II-3, both affected. A third recombination event occurred in individual III-5 who is unaffected at age 45. This recombination event would reduce the size of the region to 9 Mb, extending from marker rs1232605 to marker rs6081603. Because of the unknown penetrance of JOAG, we are using the most conservative measure for the size of the region of 12.7 Mb, which is based on the recombination events in individuals known to be affected.

To provide additional support for the new critical region, a selection of SNPs that defined the shared haplotype in JOAG-52 was evaluated in a group of smaller pedigrees (Figure 1B,C) with previous microsatellite-based haplotypes consistent with linkage to GLC1K. The affected individuals in each family shared a different haplotype, and none of the affected individuals had recombination events that led to a further reduction in the size of the GLC1K critical region.

### Evaluation of candidate genes located in the reduced GLC1K critical interval

The refined GLC1K locus lies within a gene-rich region on chromosome 20 that contains several genes that could be considered good candidates for early onset glaucoma based on their proposed function and tissue expression. Four of these genes, *BMP2*, *PLCB1*, *PLCB4*, and *BTBD3*, were selected for mutation screening. Bone morphogenetic proteins (BMPs) are multifunctional cytokines that have a broad spectrum of activities in various cell types and tissues. In retinal ganglion cells growing in culture, BMPs increased the number, length, and branch points of neurites, suggesting that BMPs function to promote and maintain ganglion cell growth [33]. Members of the BMP family have also been shown to be expressed in the human trabecular meshwork and optic nerve head [34]. *BMP2*, one member of the BMP family located within the critical region, was selected for screening because of the potential role of the protein in the development and function of both the trabecular meshwork and the retinal ganglion cells. *PLCB1* and *PLCB4* are also located within the newly defined critical region. PLCB1 has been implicated in trabecular meshwork function and has been shown to have reduced levels of expression in glaucomatous ciliary body cells grown in culture [35] while PLCB4 appears to function in the retinal visual response [36]. *BTBD3* is a member of the protein family that contains BTB/POZ domains. The BTB/POZ (broad complex Tramtrack bric-a-brac/Pox virus and zinc finger) domain is an evolutionarily conserved protein–protein interaction motif. Many BTB-containing proteins are transcriptional regulators involved in a wide range of developmental processes [37]. Several BTB/POZ homologs are involved in ocular development and retinal function. *Mri* is a drosophila homolog that was initially identified in a microarray screen for molecules that regulate retinal apoptosis, the process that retinal ganglion cells undergo in glaucoma [38]. Another BTB/POZ protein, Tramtrack69 (*Ttk69*), block neuronal photoreceptor differentiation when overexpressed [39]. The entire coding sequence of all four genes including 100 bases of the 5′ and 3′ untranslated regions and the flanking intron/exon boundaries was screened for DNA sequence variants by PCR amplification followed by direct genomic sequencing in an affected member of each family with linkage to GLC1K. Ten DNA sequence variants were detected, although none of these would be predicted to have significant biological consequences (Table 2). Studies to detect alterations of gene expression or evaluations of gene dosage were not carried out.

## Discussion

Previous studies using a collection of 25 JOAG pedigrees identified GLC1K as a juvenile-onset glaucoma locus. In the present study, using high density SNP markers and haplotype analysis, we have reduced the size of the GLC1K locus on chromosome 20 to 12.7 Mb, a reduction of over seventy percent. This reduction in size of the interval is based on recombination events in affected individuals only. If a recombination event in an unaffected individual (JOAG-52, individual III-5, age 45) is included, the size of the interval is reduced further to 9 Mb. Within the reduced region, we have screened four potential disease-causing candidate genes. Ten sequence variants were identified in JOAG-affected family members, although none of these are predicted to have a significant biological effect. Using a variety of databases (Stanford Microarray Database [40], UNIgene [41], NEIBank [42], and UCSC genome browser [31]), we have identified at least 30 genes within the refined GLC1K region, and of these genes, 24 have significant ocular expression. These genes will be prioritized for mutation screening according to the putative function of the gene product as well as tissue expression.

The improved localization of GLC1K has excluded several genes that would previously have been considered to be excellent candidates. These include a group of genes that could participate in the development of the ocular structures affected in glaucoma including *SOX12* (2.5 Mb), *EYA 2* (45 Mb), *Sall4* (49 Mb), and *VSX1* (25 Mb) [43-46]. The exclusion of *VSX1* is particularly interesting as mutations in this gene have been associated with some cases of posterior polymorphous dystrophy (PPMD), a condition that can be confused with primary juvenile open-angle glaucoma [46,47]. One locus for the oculo-oto-dental syndrome, a developmental ocular syndrome that can involve the anterior segment of the eye, has been mapped to 20q13 with peak linkage to D20S836 (44.3 Mb) [48], a location that is distal to the telomeric boundary of the refined GLC1K region, thus excluding this locus from further consideration. The reduction in size of the GLC1K region will greatly increase the efficiency of screening the remaining candidate genes located within the refined critical region.

The identification and characterization of genes responsible for early onset primary open-angle glaucoma (JOAG) may help define molecular pathways that are responsible for open-angle glaucoma including the common adult-onset form of the disease. It is also possible that a gene that is responsible for juvenile onset primary open-angle glaucoma can also contribute to adult forms of the disease as has been shown for some mutations in the gene coding for myocilin [49]. Defining the molecular mechanisms responsible for glaucoma will lead to a better understanding of the underlying pathophysiology of the disease and will lead to novel methods of treatment and diagnosis for this blinding condition.

## References

[1] Quigley HA, Broman AT (2006). The number of people with glaucoma worldwide in 2010 and 2020.. Br J Ophthalmol.

[2] Friedman DS, Wolfs RC, O’Colmain BJ, Klein BE, Taylor HR, West S, Leske MC, Mitchell P, Congdon N, Kempen J, Eye Diseases Prevalence Research Group. (2004). Prevalence of open-angle glaucoma among adults in the United States.. Arch Ophthal.

[3] Farkas RH, Grosskreutz CL (2001). Apoptosis, neuroprotection, and retinal ganglion cell death: an overview.. Int Ophthalmol Clin.

[4] Tielsch JM, Sommer A, Katz J, Royall RM, Quigley HA, Javitt J (1991). Racial variation in the prevalence of primary open-angle glaucoma. The Baltimore Eye Survey.. JAMA.

[5] Klein BE, Klein R, Sponsel WE, Franke T, Cantor LB, Martone J, Menage MJ (1992). Prevalence of glaucoma. The Beaver Dam Eye Study.. Ophthalmology.

[6] Leske MC, Connel AM, Schachat AP, Hyman L (1994). The Barbados Eye Study: prevalence of open angle glaucoma.. Arch Ophthalmol.

[7] Wiggs JL, Del Bono EA, Schauman JS, Hutchinson BT, Walton DS (1995). Clinical features of five pedigrees genetically linked to the juvenile glaucoma locus on chromosome 1q21-q31.. Ophthalmology.

[8] Weih LM, Nanjan M, McCarty CA, Taylor HR (2001). Prevalence and predictors of open-angle glaucoma: results from the visual impairment project.. Ophthalmology.

[9] Wiggs JL, Damji KF, Haines JL, Pericak-Vance MA, Allingham RR (1996). The distinction between juvenile and adult-onset primary open angle glaucoma.. Am J Hum Genet.

[10] Hewitt AW, Craig JE, Mackey DA (2006). Complex genetics of complex traits: the case of primary open-angle glaucoma.. Clin Experiment Ophthalmol.

[11] Wiggs JL (2007). Genetic etiologies of Glaucoma.. Arch Ophthal.

[12] Wang DY, Fan BJ, Chua JK, Tam PO, Leung CK, Lam DS, Pang CP (2006). A genome-wide scan maps a novel juvenile-onset primary open angle glaucoma locus to 15q.. Invest Ophthalmol Vis Sci.

[13] Fan BJ, Wang DY, Lam DS, Pang CP (2006). Gene mapping for primary open angle glaucoma.. Clin Biochem.

[14] Wiggs JL, Allingham RR, Hossain A, Kern J, Auguste J, DelBono EA, Broomer B, Graham FL, Hauser M, Pericak-Vance M, Haines JL (2000). Genome-wide scan for adult-onset primary open angle glaucoma.. Hum Mol Genet.

[15] Stone EM, Fingert JH, Alward WL, Nguyen TD, Polansky JR, Sunden SL, Nishimura D, Clark AF, Nystuen A, Nichols BE, Mackey DA, Ritch R, Kalenak JW, Craven ER, Sheffield VC (1997). Identification of a gene that causes primary open angle glaucoma.. Science.

[16] Rezaie T, Child A, Hitchings R, Brice G, Miller L, Coca-Prados M, Heon E, Krupin T, Ritch R, Kreutzer D, Crick RP, Sarfarazi M (2002). Adult-onset primary open-angle glaucoma caused by mutations in optineurin.. Science.

[17] Monemi S, Spaeth G, DaSilva A, Popinchalk S, Ilitchev E, Liebmann J, Ritch R, Heon E, Crick RP, Child A, Sarfarazi M (2005). Identification of a novel adult-onset primary open angle glaucoma (POAG) gene on 5q22.1.. Hum Mol Genet.

[18] Hauser MA, Allingham RR, Linkroum K, Wang J, LaRocque-Abramson K, Figueiredo D, Santiago-Turla C, del Bono EA, Haines JL, Pericak-Vance MA, Wiggs JL (2006). Distribution of WDR36 DNA sequence variants in patients with primary open-angle glaucoma.. Invest Ophthalmol Vis Sci.

[19] Wiggs JL, Allingham RR, Vollrath D, Jones KH, De La Paz M, Kern J, Patterson K, Babb VL, Del Bono EA, Broomer BW, Pericak-Vance MA, Haines JL (1998). Prevalence of mutations in TIGR/Myocilin in patients with adult and juvenile primary open-angle glaucoma.. Am J Hum Genet.

[20] Fingert JH, Heon E, Liebman JM, Yamamoto T, Craig JE, Rait J, Kawase K, Hoh ST, Buys YM, Dickinson J, Hockey RR, Williams-Lyn D, Trope G, Kitazawa Y, Ritch R, Mackey DA, Alward WL, Sheffield VC, Stone EM (1999). Analysis of myocilin mutations in 1703 glaucoma patients from five different populations.. Hum Mol Genet.

[21] Bruttini M, Longo I, Frezzoti P, Ciappetta R, Randazzo A, Orzalesi N, Fumagalli E, Caporossi A, Frezzotti R, Renieri A (2003). Mutations in the myocilin gene in families with primary open-angle glaucoma and juvenile open-angle glaucoma.. Arch Ophthalmol.

[22] Alward WL, Kwon YH, Kawase K, Craig JE, Hayreh SS, Johnson AT, Khanna CL, Yamamoto T, Mackey DA, Roos BR, Affatigato LM, Sheffield VC, Stone EM (2003). Evaluation of optineurin sequence variations in 1,048 patients with open-angle glaucoma.. Am J Ophthalmol.

[23] Wiggs JL, Auguste J, Allingham RR, Flor JD, Pericak-Vance MA, Rogers K, LaRocque KR, Graham FL, Broomer B, Del Bono E, Haines JL, Hauser M (2003). Lack of association of mutations in optineurin with disease in patients with adult-onset primary open-angle glaucoma.. Arch Ophthal.

[24] Tang S, Toda Y, Kashiwagi K, Mabuchi F, Iijima H, Tsukahara S, Yamagata Z (2003). The association between Japanese primary open-angle glaucoma and normal tension glaucoma patients and the optineurin gene.. Hum Genet.

[25] Leung YF, Fan BJ, Lam DS, Lee WS, Tam PO, Chua JK, Tham CC, Lai JS, Fan DS, Pang CP (2003). Different optineurin mutation pattern in primary open-angle glaucoma.. Invest Ophthalmol Vis Sci.

[26] Wiggs JL, Lynch S, Ynagi G, Maselli M, Auguste J, Del Bono EA, Olson LM, Haines JL (2004). A genomewide scan identifies novel early-onset primary open angle glaucoma loci on 9q22 and 20p12.. Am J Hum Genet.

[27] Sheffield VC, Stone EM, Alward WL, Drack AV, Johnson AT, Streb LM, Nichols BE (1993). Genetic linkage of familial open angle glaucoma to chromosome 1q21-q31.. Nat Genet.

[28] Lin Y, Liu T, Li J, Yang J, Du Q, Wang J, Yang Y, Liu X, Fan Y, Lu F, Chen Y, Pu Y, Zhang K, He X, Yang Z (2008). A genome-wide scan maps a novel autosomal dominant juvenile-onset open-angle glaucoma locus to 2p15–16.. Mol Vis.

[29] De La Vega FM, Isaac HI, Scafe CR (2006). A tool for selecting SNPs for association studies based on observed linkage disequilibrium patterns.. Pac Symp Biocomput.

[30] Sobel E, Sengul H, Weeks DE (2001). Multipoint estimation of identity-by-descent probabilities at arbitrary positions among marker loci on general pedigrees.. Hum Hered.

[31] Kent WJ, Sugnet CW, Furey TS, Roskin KM, Pringle TH, Zahler AM, Haussler D (2002). The Human Genome Browser at UCSC.. Genome Res.

[32] International Human Genome Sequencing Consortium (2001). Initial sequencing and analysis of the human genome.. Nature.

[33] Kerrison JB, Lewis RN, Otteson DC, Zack DJ (2005). Bone morphogenetic proteins promote neurite outgrowth in retinal ganglion cells.. Mol Vis.

[34] Wordinger RJ, Agarwal R, Talati M, Fuller J, Lambert W, Clark AF (2002). Expression of bone morphogenetic proteins (BMP), BMP receptors, and BMP associated proteins in human trabecular meshwork and optic nerve head cells and tissues.. Mol Vis.

[35] Husain S, Kaddour-Djebbar I, Abdel-Latif AA (2002). Alterations in arachidonic acid release and phospholipase C-beta(1) expression in glaucomatous human ciliary muscle cells.. Invest Ophthalmol Vis Sci.

[36] Jiang H, Lyubarsky A, Dodd R, Vardi N, Pugh E, Baylor D, Simon MI, Wu D (1996). Phospholipase C beta 4 is involved in modulating the visual response in mice.. Proc Natl Acad Sci USA.

[37] Collins T, Stone JR, Williams AJ (2001). All in the family: the BTB/POZ, KRAB, and SCAN domains.. Mol Cell Biol.

[38] Rusconi JC, Challa U (2007). Drosophila Mrityu encodes a BTB/POZ domain-containing protein and is expressed dynamically during development.. Int J Dev Biol.

[39] Wen Y, Nguyen D, Li Y, Lai ZC (2000). The N-terminal BTB/POZ domain and C-terminal sequences are essential for Tramtrack69 to specify cell fate in the developing Drosophila eye.. Genetics.

[40] Marinelli RJ, Montgomery K, Liu CL, Shah NH, Prapong W, Nitzberg M, Zachariah ZK, Sherlock GJ, Natkunam Y, West RB, van de Rijn M, Brown PO, Ball CA (2008). The Stanford Tissue Microarray Database.. Nucleic Acids Res.

[41] Wheeler DL, Barrett T, Benson DA, Bryant SH, Canese K, Chetvernin V, Church DM, Dicuccio M, Edgar R, Federhen S, Feolo M, Geer LY, Helmberg W, Kapustin Y, Khovayko O, Landsman D, Lipman DJ, Madden TL, Maglott DR, Miller V, Ostell J, Pruitt KD, Schuler GD, Shumway M, Sequeira E, Sherry ST, Sirotkin K, Souvorov A, Starchenko G, Tatusov RL, Tatusova TA, Wagner L, Yaschenko E (2008). Database resources of the National Center for Biotechnology Information.. Nucleic Acids Res.

[42] Wistow G (2006). The NEIBank project for ocular genomics: data-mining gene expression in human and rodent eye tissues.. Prog Retin Eye Res.

[43] Dy P, Penzo-Méndez A, Wang H, Pedraza CE, Macklin WB, Lefebvre V (2008). The three SoxC proteins–Sox4, Sox11 and Sox12–exhibit overlapping expression patterns and molecular properties.. Nucleic Acids Res.

[44] Ishihara T, Ikeda K, Sato S, Yajima H, Kawakami K (2008). Differential expression of Eya1 and Eya2 during chick early embryonic development.. Gene Expr Patterns.

[45] Harvey SA, Logan MP (2006). sall4 acts downstream of tbx5 and is required for pectoral fin outgrowth.. Development.

[46] Héon E, Greenberg A, Kopp KK, Rootman D, Vincent AL, Billingsley G, Priston M, Dorval KM, Chow RL, McInnes RR, Heathcote G, Westall C, Sutphin JE, Semina E, Bremner R, Stone EM (2002). VSX1: a gene for posterior polymorphous dystrophy and keratoconus.. Hum Mol Genet.

[47] Shimizu S, Krafchak C, Fuse N, Epstein MP, Schteingart MT, Sugar A, Eibschitz-Tsimhoni M, Downs CA, Rozsa F, Trager EH, Reed DM, Boehnke M, Moroi SE, Richards JE (2004). A locus for posterior polymorphous corneal dystrophy (PPCD3) maps to chromosome 10.. Am J Med Genet A.

[48] Vieira H, Gregory-Evans K, Lim N, Brookes JL, Brueton LA, Gregory-Evans CY (2002). First genomic localization of oculo-oto-dental syndrome with linkage to chromosome 20q13.1.. Invest Ophthalmol Vis Sci.

[49] Hewitt AW, Mackey DA, Craig JE (2008). Myocilin allele-specific glaucoma phenotype database.. Hum Mutat.

